# Biomimetics of Bone Implants: The Regenerative Road

**DOI:** 10.1089/biores.2016.0044

**Published:** 2017-01-01

**Authors:** Elizabeth Brett, John Flacco, Charles Blackshear, Michael T. Longaker, Derrick C. Wan

**Affiliations:** ^1^ Hagey Laboratory for Pediatric Regenerative Medicine, Plastic and Reconstructive Surgery Division, Department of Surgery, Stanford University School of Medicine, Stanford, California.; ^2^ Institute for Stem Cell Biology and Regenerative Medicine, Stanford University, Stanford, California.

**Keywords:** biomimetic, bone graft, implant, osteoconduction, osteoinduction, stem cell

## Abstract

The current strategies for healing bone defects are numerous and varied. At the core of each bone healing therapy is a biomimetic mechanism, which works to enhance bone growth. These range from porous scaffolds, bone mineral usage, collagen, and glycosaminoglycan substitutes to transplanted cell populations. Bone defects face a range of difficulty in their healing, given the composite of dense outer compact bone and blood-rich inner trabecular bone. As such, the tissue possesses a number of inherent characteristics, which may be clinically harnessed as promoters of bone healing. These include mechanical characteristics, mineral composition, native collagen content, and cellular fraction of bone. This review charts multiple biomimetic strategies to help heal bony defects in large and small osseous injury sites, with a special focus on cell transplantation.

## Introduction

Biomimetics and biomimicry are thought processes applied to biomaterial design, where materials meant for implantation have properties, which mirror closely those of natural material. Design of implants in this manner can circumvent some of the roadblocks in synthetic biomaterial design and function.^1^ This review will highlight the variety of biomimetic biomaterial research, with a specific focus on bone regeneration.

Clinically, a biomaterial needs to predictably accomplish its role 100% of the time. In recent years, biomimetic R&D has identified issues with existing materials and focused on aspects of implants such as physical shape, surface chemistry, and mechanical properties. Deleterious outcomes of implants, biologic or synthetic (peri-implantitis, peri-mucositis, peri-implant disease, infection), can mean pain, mechanical loosening, failure, and eventual need for extraction.^2,3^ Similarly, some literature has outlined concerns with implants, specifically showing unfavorable physical remodeling over time.^4^ Eliminating variability of results in biomaterial implantation means a huge decrease in patient morbidity and associated costs. These concerns have ushered biomimetics and new material design techniques to the forefront.

Bone is one of the few tissues capable of complete regeneration in adult humans. As such, bone healing has several distinct aspects of healing, which translate very well to biomimetic biomaterial design. Osteoconductive scaffolds, osteoinductive stimuli, and potent osteogenic cell populations are options for a therapy, in which cells, extracellular matrices (ECM), and chemical signaling act in concert to rapidly heal bone.^5^

**FIG. 1.**
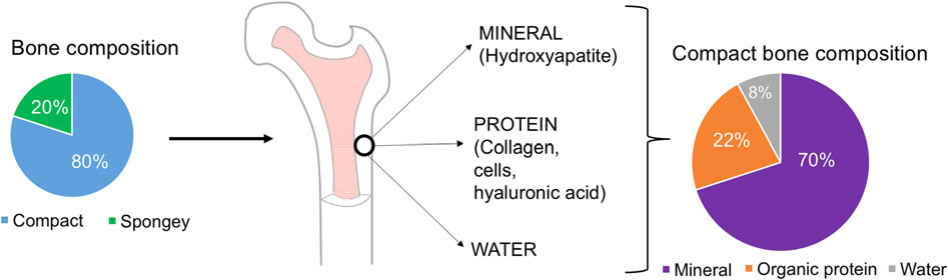
Schematic showing composition of bone between compact (cortical) and spongy (cancellous). Cortical bone is evaluated further and broken into its constituents; 70% mineral, 22% organic protein, 8% water.

It is important to consider the material breakdown of bone to best replace like with like. Eighty percent of bone is made up of the outer, compact (cortical) bone, while 20% remaining is inner, spongy (trabecular) bone.^6^ Therefore, analyzing the content of compact bone will focus the therapy on healing the larger portion of the bone. Knowing that compact bone is 70% inorganic mineral (chiefly hydroxyapatite), 22% organic protein (collagen, cells, hyaluronic acid [HA]), and 8% water allows for bone graft design to focus on one compartment^7^ (Fig. 1).

## The Recipient Site

Types of bony reconstruction range from structural nonload-bearing bone (e.g., calvarium) to larger compact load-bearing bone (e.g., femur). Host bone can often present an orthopedic challenge. Osteoporotic bone will be a weak tissue to host implant hardware.^8^ Similarly, osteomyelitic bone is a major contraindication for implants, considering the likelihood of infectious staphylococci biofilm formation.^9,10^ To ensure a fit of the implant in the host bone, under-reaming is often performed. This is a drilling process designed to facilitate the implant, which is sometimes known to fracture the host bone further.^11^ It is especially problematic in the context of osteoporotic bone, which can exhibit low “pull out strength” of an implant once *in situ* due to insufficient cortical bone.^12^ Quite common also is the issue of poor nutrient diffusion, borne of compromised blood supply. Diffusion distance of oxygen *in vivo* is 150–200 μm.^13^ As such, certain materials operate via stimulation of vascular ingrowth into the host bone. Use of cobalt ions as inactivators of prolyl hydroxylase has been shown to stabilize *HIF-1α*, a potent proangiogenic factor, resulting in upregulated expression of genes such as *GLUT1*, erythropoietin, *VEGF*, and *PDGF*.^14,15^ Bone that has undergone irradiation is an example of a hostile recipient site. There is conflicting retrospective literature, which debates the use of hyperbaric oxygen (HBO) in treating host bone, specifically during craniofacial reconstruction after head and neck cancer. Some studies show improved vascularity and increased basic fibroblast growth factor of grafted recipient bone,^16^ and other studies exhibit no significant difference in graft take with HBO.^17^ Macroscopically, there is reliance on mechanical design of threaded implants to give the implant mechanical integration. The ability to screw an implant through bone gives immediate stability and provides close, fixed contact between the graft and host bone.

## Mechanical Stability of Implant

Issues with bone implants chiefly lie in materials destined for load-bearing bone healing. The Young's modulus of trabecular bone is 10.4–14.8 GPa, and cortical bone measures 18.4–20.7 GPa.^18^ These values differ drastically from those of commonly used metals for bone implants; stainless steel measuring 180 GPa, cobalt chromium (Co-Cr) at 210 GPa, and titanium at 110 GPa.^19^ Stress shielding is the physiological response resulting from implanting a harder material into a softer host tissue. The physiological result manifests in fibrous encapsulation of implant where possible, allowing for micromovement of the fibrotic sheath around the implant. Mobility of an implant *in situ* on the order <100 μm creates a specific wear, called fretting.^20^ Fretting implants can gradually loosen and eventually fail within the host bone.^21^ Moreover, fragments of the implant are frequently known to break off the body of the implant, causing local abrasion of the surrounding, softer bone tissue. Macrophages have been seen to internalize particles of polyethyleneimine surrounding total hip arthroplasties.^22^ Furthermore, debris of implants can sometimes be found in alternate locations in the body, such as the spleen, liver, and abdominal lymph nodes of arthroplasty patients.^23^

It was Branemark in 1981 who first showed the complete “osseointegration” of titanium. During a fracture fixation experiment in a rabbit femur, Branemark discovered the removal of the titanium implant from bone was impossible. Further studies using transmission electron microscopy showed direct contact between bone and implant, without the surrounding fibrous capsule responsible for implant looseness and micromovement.^24^ Filamentous collagen type 1 was found to form fibrils at the implant–bone interface resembling the strong Sharpey's fibers of the scalp, explaining the tight coupling of metal and bone.^25^ The phenomenon of the absence of fibrous encapsulation around titanium implants has proven significantly important in implant design in more than just bone. Tissue expanders used for breast reconstruction can include a titanium-coated mesh to reduce the fibrotic content of the breast.^26^ In the context of bone, titanium is now being tested in advanced models of craniofacial bone healing, using porous titanium granules as new-bone regeneration stimuli to recreate maxillary sinuses.^27^ However, in terms of biomimetics, titanium presents some mechanical challenges, specifically in terms of difference in stiffness between it and the host tissue, for example, femur. This hurdle is called modular mismatch^28^ and can be circumvented by utilizing material whose bulk mechanical properties more closely resemble bone.

## Grafts Based on Bone Mineral Components

The unique nature of bone is that it is largely mineralized. In its dry mass, bone is 60–70% mineral, which is nonimmunogenic and ubiquitously found.^29^ As such, the use of natural material already in existence presents an option for creating biomimetic implant matter. Nacre, or mother-of-pearl, is pure calcium carbonate produced by molluscs. Mixing pulverized nacre with patient blood and implanting the mixture into a human mandibular defect site was largely effective in closing the defect and stimulating regenerative cellular activity in the location.^30^ Many successful bone biomaterials incorporate calcium or hydroxyapatite to help facilitate bone formation and graft “take.” A study comparing biologic bovine-derived bone grafts (BioOss^®^ Bone Substitute; Ed. Geistlich Soehne, Wolhusen, Switzerland), with a highly porous synthetic hydroxyapatite scaffold (IngeniOs™ HA Synthetic Bone Particles; Zimmer Dental, Inc., Carlsbad, CA), showed highly similar chemistry, morphology, and structure with the exception of crystallinity.^27^ FT-IR spectra revealed high crystallinity (thus low resorption) of the synthetic IngeniOs Hydroxyapatite Synthetic Bone Particles, owing to the purity of its manufacturing processes versus natural variation and other trace elements inherent to biologic grafts.^31^ The difference in purity between synthetic and biologic material by proxy of uniform industrial manufacture is seen throughout most implant forms.

Beta tricalcium phosphate (β-TCP) is a calcium salt abundant in bone, and has been shown to be highly and quickly reactive as part of a bone graft. This is because in an aqueous environment, TCP reacts to form hydroxyapatite.^32^ Effects of TCP can be exaggerated with strategic addition of growth factors *in vivo*, as was seen throughout a series of randomized control trials using platelet-derived growth factor and β-TCP as agents to heal periodontal intraosseous defects.^33^ In fact, β-TCP has been shown to contribute to bone healing faster than hydroxyapatite alone, secondary to its rapid rate of resorption.^34^ Meanwhile, HAPEX is an amalgamation of the biologic mineral hydroxyapatite and synthetic high-weight polyethylene. This mixture makes for a bioactive polymer, which has been used in the reconstruction of orbital floor and middle ear defects.^35^

The ideal bone implant will resorb completely after a time of osteoinduction or osteoconduction of surrounding healing tissue. Most bone healing substitutes today incorporate a biologic component, intended to mimic native bone mechanical structure or mineral chemistry. Cerasorb is pure β-TCP, designed to be mixed with the patient's own blood or platelet-rich plasma and added to the defect site, primarily for periodontic healing (http://curasaninc.com/products/cerasorb). Alternatively, natural bone biomimetics have been found in coral, for both chemistry and structure. Coral forms hydroxyapatite on its surface due to its calcium carbonate core and also retains its native trabecular structure, having inherent biomedical value as a spongy bone substitute.^36,37^

## Grafts Based on Structural Protein

To further the biomimetic approach of bone grafts, materials are being designed with a microscopic eye on ECM. These incorporate more biologic factors than synthetic, such as endogenous protein, collagen, and HA. HA is a high-molecular-weight nonsulfated glycosaminoglycan, which is formed in the plasma membrane of cells.^38^ Many biomimetic ECM scaffolds containing HA have reached the market for dermal applications, showing hyaluronate as an antifibrotic hydrating agent in a healing wound.^39^ However, in the context of bone grafts, rabbit tibias, which received HA, showed increased healing 20 days after injury, showing fibrocartilage formation, which later ossified, in comparison to the non-HA-treated bones, which formed purely fibrous unions.^40^ More sophisticated, combinatorial approaches have been performed, involving collagenase to stimulate mandibular bone remodeling, and HA in a hydrogel with calcium sulfide hemihydrate; a bioresorbable, osteoconductive compound.^41^

Composite biomimetic grafts over simple hydrogel injection are required for healing of large load-bearing bones. For instance, addition of protein to a hydrogel or impregnated into an implant introduces cellular osteogenic mechanisms. In a canine femoral defect model, bone morphogenetic protein (BMP) in tandem with collagen type-1/TCP showed increased healing of the femur in the presence of bone marrow aspirate.^42^ This study points to the use of collagen type 1 to match the modulus of the femur, and the utility of bone marrow-derived cells stimulated by pro-osteogenic growth factors in bone healing.

A highly osteogenic component of bone is the periosteum, a stratified structure of an inner cell layer (cambium layer), and tough, fibrous outer layer.^43^ In fact, damaged bone, which undergoes delayed reconstruction, often exhibits heterotopic ossification; new disorganized bone formation at the injury site as a result of damaged periosteum.^44^ As such, strategically placed periosteal grafts present a highly biomimetic solution of autografting onto damaged bone.^45^ However, due to the sheet-like structure of the periosteum, periosteal grafts have found their utility primarily in dental and alveolar healing, as opposed to long, load-bearing bone reconstruction.^46^

## Grafts Based on Cellular Implants

Despite orthopedic management of fractures becoming better and better, some healed injuries will persist with fibrous nonunions,^47^ an issue that may still be encountered clinically. To address this, “The Diamond Concept” has been reported, which encompasses four different aspects of *in vivo* bone regeneration; an osteoconductive scaffold, a suitable mechanical environment, osteoinductive signals, and a pro-osteogenic cell population.^5^

The ability to direct stem cells to an osteogenic pathway represents a huge regenerative medical role. Addition of cells to bone grafts is a concept based on isolating cell types, which will either immediately and directly add bone and aid in the remodeling procedure (osteoblasts/osteoclasts), or have the potential to differentiate, affording the healing tissue with angiogenic and osteogenic factors (mesenchymal stem cells [MSCs] and adipose-derived stem cells [ASCs]).^48^ Moreover, there are now established surface protein expression profiles to identify heterogeneous stem cells, which are more likely to differentiate into bone.

Cell surface markers indicative of osteogenic behavior can vary from poorly defined CD markers to more well-known pro-osteogenic growth factor receptors. BMP receptor type-1b (BMPR-1b) binds BMP and has an important role in directing bone formation.^49^ As such, using FACS to select for BMPR-1b-positive cells from ASCs results in a population with increased osteogenic gene expression and *in vitro* osteogenic potential.^50^ Moreover, when coupled with a porous, osteoconductive scaffold coated in osteoinductive hydroxyapatite, rapid bone formation is observed *in vivo.*^51^ Similarly, FACS sorted ASCs positive for CD90 (Thy-1) have been shown to significantly increase healing of bone defects when compared with their negative and unsorted control groups.^52,53^ Importantly, CD90 expression has been shown to vary dramatically between *in vivo* and *in vitro* settings, making use of this marker unpredictable.^54^ Similarly, other markers also have difficult expression profiles to track and analyze, such as CD105 (endoglin), a bone marrow mesenchymal cell marker. Expression of CD105 in freshly harvested ASCs is extremely low, proceeded by near ubiquitous expression after 4–7 days in culture. However, it was discovered that isolating CD105-negative cells after 36 h in culture yielded a subpopulation with enhanced osteogenic potential *in vivo.*^55^ CD105 is especially nuanced, although in that it acts as a coreceptor for TGF-β1, which is a known antagonist of osteogenic differentiation,^56^ thus, explaining a parallel decrease in bone formation with increased expression. These findings simultaneously highlight the promise and problems of using surface marker selection criteria in isolating heterogeneous stem cell populations for bone regenerative purposes.

There are multiple different bone diseases being researched under an autologous cell transfer lens. For instance, the efficacy of bone regeneration by autologous bone marrow harvested from the anterior iliac crest has been shown in atrophic diaphyseal nonunion. Here, a biomaterial was created by concentrating marrow via centrifugation, which could be loaded into a syringe and injected into the recipient site. Analysis of diseased tibias postmarrow transplant showed increased bone callus mineralization.^57^ Similarly, osteogenesis imperfecta (OI) is a genetic disease of the mesenchymal cells, whereby a defective collagen type 1 is produced, giving rise to bone weakness and malformation. Unmanipulated bone marrow donations from healthy matched siblings or family members have been shown to increase trabecular bone formation in the recipient OI patient.^58^ On the cellular level, the harvest, culture, and transplant of bone marrow-derived stromal cells have been found to be effective in repairing large bone defects in humans.^59^ However, these cells are most efficient when placed *in situ* seeded on a macroporous scaffold.^60^

Combinations of cell populations with scaffolds have shown efficacy in healing bone defects. It was seen in composite grafts of BMP-2, HA-based hydrogel, and hMSCs that there was a synergistic effect of the group, which received cells over those that did not, in the healing of rat calvarial defects.^61^ In a human pediatric case study, autologous ASCs used with autologous fibrin glue (derived from patient's own blood) were used successfully together to heal the patient's widespread calvarial defects.^62^ From an endochondral ossification perspective, cell culture offers a biomimetic tissue engineering route. Culturing chondrocytes on a porous polylactic acid scaffold revealed deposition of collagen type II and glycosaminoglycans, mimicking the *in vivo* histology of cartilage.^63^ The use of specialized cell populations grown on scaffolds can be a future strategic method of stimulating bone/cartilage formation *in vivo*.

## Conclusion

To meet key requirements of bone regeneration, we need to move beyond the current standards of therapies and toward regenerative strategies. Biomimetics allows us to learn from and emulate the inherently self-sufficient healing mechanisms already in place. Due to the biological nature of much of the research mentioned in this review, product development still faces considerable regulatory hurdles. However, the knowledge bank, which is borne of this research, has allowed extremely effective biologic treatments to be developed. The biomimetic boundaries that can be pushed belong to implanted biological matter; collagen or collagen analogs, soluble minerals, or active cell populations.

## Abbreviations Used

β-TCP: beta tricalcium phosphate
ASC: adipose-derived stem cell
BMP: bone morphogenetic protein
BMPR-1b: Bone morphogenetic protein receptor type-1b
ECM: extracellular matrices
HA: hyaluronic acid
HBO: hyperbaric oxygen
MSCs: mesenchymal stem cells
OI: osteogenesis imperfecta
